# Benefit analysis of the auto-verification system of intelligent inspection for microorganisms

**DOI:** 10.3389/fmicb.2024.1334897

**Published:** 2024-03-18

**Authors:** Yu-Hsiang Ou, Yung-Ta Chang, Ding-Ping Chen, Chun-Wei Chuang, Kuo-Chien Tsao, Chiu-Hsiang Wu, An-Jing Kuo, Huey-Ling You, Chung-Guei Huang

**Affiliations:** ^1^Department of Laboratory Medicine, Linkou Chang Gung Memorial Hospital, Taoyuan, Taiwan; ^2^Department of Medical Biotechnology and Laboratory Science, College of Medicine, Chang Gung University, Taoyuan, Taiwan; ^3^Graduate Institute of Biomedical Sciences, College of Medicine, Chang, Gung University, Taoyuan,, Taiwan; ^4^Department of Laboratory Medicine, Chang Gung Memorial Hospital, Keelung, Taiwan; ^5^Departments of Laboratory Medicine, Kaohsiung Chang Gung Memorial Hospital, Kaohsiung, Taiwan

**Keywords:** microbiology, auto-verification, turnaround time, matrix-assisted laser desorption/ionization-time of flight mass spectrometer, microorganisms

## Abstract

In recent years, the automatic machine for microbial identification and antibiotic susceptibility tests has been introduced into the microbiology laboratory of our hospital, but there are still many steps that need manual operation. The purpose of this study was to establish an auto-verification system for bacterial naming to improve the turnaround time (TAT) and reduce the burden on clinical laboratory technologists. After the basic interpretation of the gram staining results of microorganisms, the appearance of strain growth, etc., the 9 rules were formulated by the laboratory technologists specialized in microbiology for auto-verification of bacterial naming. The results showed that among 70,044 reports, the average pass rate of auto-verification was 68.2%, and the reason for the failure of auto-verification was further evaluated. It was found that the main causes reason the inconsistency between identification results and strain appearance rationality, the normal flora in the respiratory tract and urine that was identified, the identification limitation of the mass spectrometer, and so on. The average TAT for the preliminary report of bacterial naming was 35.2 h before, which was reduced to 31.9 h after auto-verification. In summary, after auto-verification, the laboratory could replace nearly 2/3 of manual verification and issuance of reports, reducing the daily workload of medical laboratory technologists by about 2 h. Moreover, the TAT on the preliminary identification report was reduced by 3.3 h on average, which could provide treatment evidence for clinicians in advance.

## 1 Introduction

Intelligent inspection has become the modern mainstream. In recent years, various microbiology laboratories have successively introduced automated machines for inoculation or identification of microbial specimens, antibiotic susceptibility testing (AST), and even the emergence of comprehensive laboratory automation for microbiology (Antonios et al., 2021). However, there are still many inspection steps that require manual operation before the full-scale automation track goes online, such as specimen receipt, specimen pretreatment, culture medium interpretation, bacterial name report verification, antibiotic susceptibility test report approval, etc., and the issuance of reports is an important part of complicated operations.

In the testing process, the purpose of various process optimizations is to shorten the timeliness of laboratory reports to provide clinicians with the fastest and correct test data for clinical diagnosis and treatment. A study showed that when the outlier percentage of turnaround time (TAT) in the laboratory was reduced from 14.4 to 4.9%, the hospital length of stay (LOS) in the emergency room could be reduced from 4.1 h to 3.2 h (Holland et al., 2005). Another study also confirmed that the reduction of TAT in the laboratory could indeed reduce the time patients stay in the emergency room (Kaushik et al., 2018). The analysis of patients with unknown pneumonia found that the time from admission to the first report of microbiological results was significantly related to hospital LOS (Shrestha et al., 2022). Although there are many variables in the period from hospitalization to the first report of microbiological results, optimizing the laboratory TAT is a very clear direction and choice. Research also indicates that the implementation of Total Laboratory Automation (TLA) systems can reduce sample processing time, optimize workflow, and decrease TAT. Furthermore, a significant reduction in the culture time of cerebrospinal fluid (CSF) samples has been observed, allowing for timely initiation of antibiotic treatment, and leading to a decrease in hospitalization costs and mortality rates (Zhang et al., 2021). Currently, in the field of clinical microbiology, there is a lack of research exploring the automatic verification of microbial species using MALDI-TOF. Therefore, we developed a unique verification system to optimize the efficiency of MALDI-TOF utilization. It is very important to continuously ameliorate the medical laboratory testing process in clinical testing to improve the quality of medical care.

Recently, the auto-verification system has been widely used in blood, biochemical, and other groups. In addition, the specifications of the American Clinical & Laboratory Standards Institute (CLSI), AUTO 10-A (Neeley et al., 2006), and AUTO-15 (CLSI, 2019), clearly define how to design, implement and ensure the effectiveness of the auto-verification system. Previous research (Randell et al., 2019; Wang et al., 2019) have indicated that auto-verification could reduce the error rate (Torke et al., 2005; Lin et al., 2019), ensure medical safety (Wu et al., 2018), shorten the reporting time (Torke et al., 2005; Sediq and Abdel-Azeez, 2014; Li et al., 2018; Wu et al., 2018), reduce the manpower demand (Wongkrajang et al., 2020), and improve work efficiency (Torke et al., 2005; Sediq and Abdel-Azeez, 2014; Li et al., 2018; Randell et al., 2018a, 2018b). However, these auto-verification systems are usually developed through third-party commercial software, which is costly. Additionally, the decision rules of auto-verification are proprietary, making them non-modifiable according to user requirements (Gomez-Rioja et al., 2013; Jones, 2013; Li et al., 2016; Randell et al., 2018a, 2018b; Gul et al., 2022). Although auto-verification has many advantages, not all medical laboratories are suitable for auto-verification by themselves. In the CLSI AUTO-15 specification, it was pointed out that blood transfusion medicine, microbiology, molecular medicine, anatomical pathology, or point-of-care testing (POCT) does not apply to this specification. However, because most microbiological tests are qualitative tests, there are no regulations that clearly explain how such medical laboratories perform auto-verification.

After the popularization of various automatic machines for microbial inspection, there have been study on the auto-verification of microbial molecular inspection reports (Durant et al., 2019; Cai et al., 2023), and the TAT has been significantly reduced. However, at present, there is no traditional microbial laboratory for the auto-verification of bacterial name reports. Traditional microbiological testing can rapidly improve the TAT after being introduced into the mass spectrometry identification system. It can be shortened from 1 to 2 days to several hours or even minutes to complete accurate bacterial identifications. In addition, it can also be used as a sharp tool in clinical research, such as the analysis and prediction of drug-resistant strains using the mass spectrometer (Weis et al., 2022; Moreira et al., 2023), and the direct extraction method of positive blood culture (Azrad et al., 2019; Dai et al., 2021). The ultimate goal of these studies is to shorten the TAT. Although the identification system of the microbiological laboratory can provide the identification results quickly, it still requires labor and time costs to confirm and verify the reports one by one. Moreover, the microbiological test is a manual interpretation of the culture medium and is a qualitative result. There is no fixed reference value to set the abnormal value, nor can it accurately perform the delta checking according to CLSI specifications. Therefore, additional research and discussion are required for the auto-verification logic design of the microbiology laboratory. Consequently, the purpose of this study was to establish an auto-verification system for the preliminary report of microbial names and to verify the preliminary report of microbial names identified by a mass spectrometer to improve the efficiency of the preliminary identification report and reduce the burden of clinical laboratory technologists.

## 2 Materials and methods

### 2.1 Source of subjects

In this study, the positive strains of the specimens cultured in the microbiology laboratory of Linkou Chang Gung Memorial Hospital from January 2021 to February 2022 were used for colony identification and subsequent analysis.

### 2.2 Microbiological examination process

**Figure 1:**
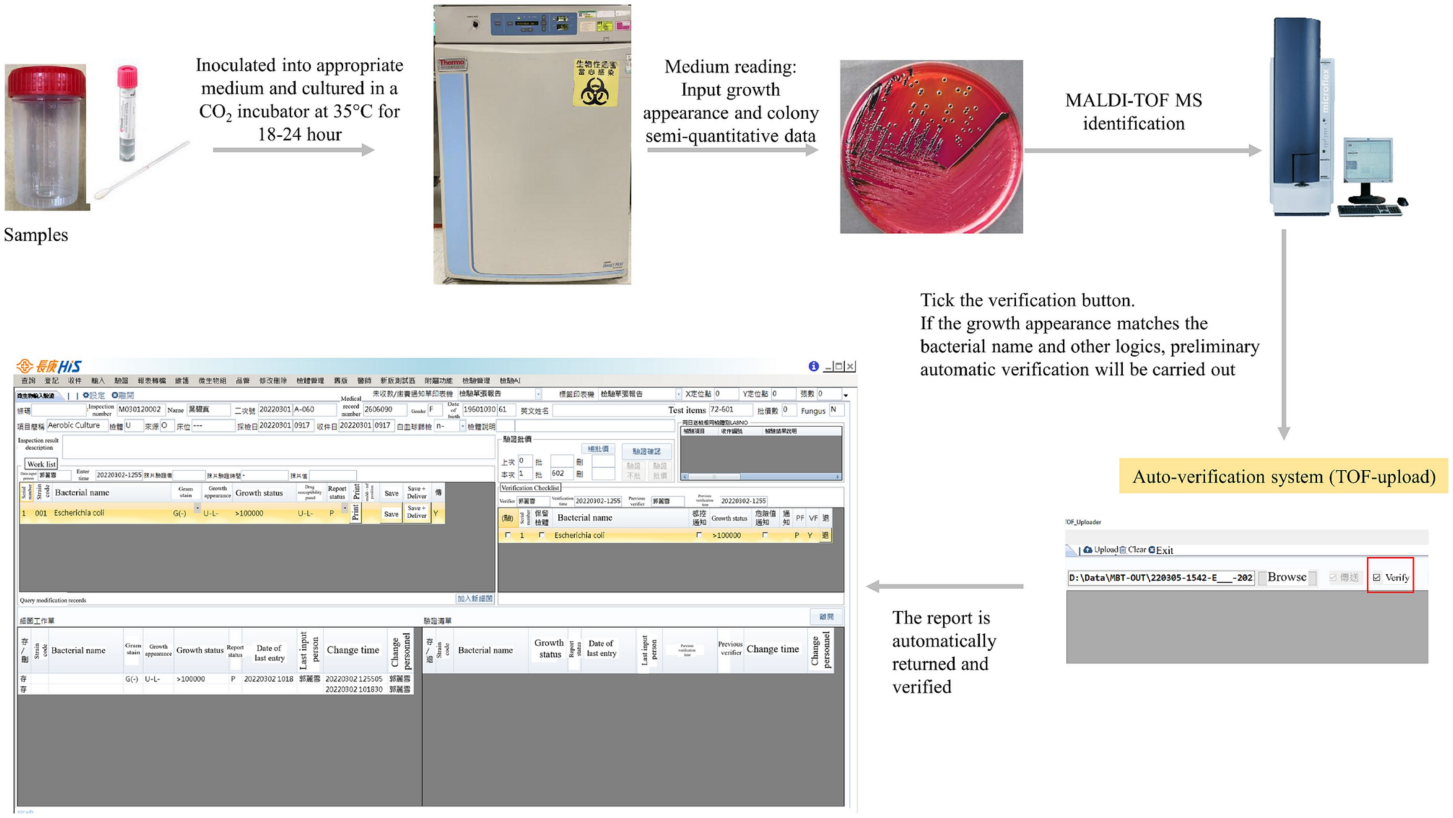
The flow chart of the microbiological examination process.

The sources of specimens in this laboratory include urine [including urine, catheterized urine, percutaneous nephrostomy (PCN), suprapubic aspirate (SPA)], respiratory tract (including sputum, bronchial washing, bronchial-alveolar lavage), wound (including wound, pus, abscess), tissue, bile, drainage fluid, catheter (CVP tip), body fluids (including ascites, synovial fluid, pleural effusion, CSF, pericardial fluid, amniotic fluid, dialysate), and others (including nasopharyngeal swab, conjunctival swab, corneal ulcer, bone, cervix discharge, endocervix discharge). The specimens were inoculated into the appropriate medium and cultured in a 5% CO_2_ incubator at 35°C for 18–24 h for aerobic bacterial culture. Then, the culture medium was interpreted, and the potentially pathogenic bacteria were selected. The information such as Gram’s staining result, semi-quantitative quantity, strain growth appearance, and report status was sequentially input in the laboratory information system (LIS), and then the colony identification and analysis were carried out by the matrix-assisted laser desorption time-of-flight mass spectrometry (MALDI-TOF MS). After the identification was completed, the identification results would be uploaded by using the TOF-upload system developed by our institute. If the identification score of MALDI-TOF MS was ≥2.0 and met the auto-verification rules, the preliminary auto-verification of the bacterial naming would be completed. If it did not meet the rules, only the bacterial name would be uploaded without auto-verification, and the identification results would be manually checked on behalf of the following. The flow chart was shown in Figure 1.

### 2.3 Logic designs of the auto-verification

**Figure 2:**
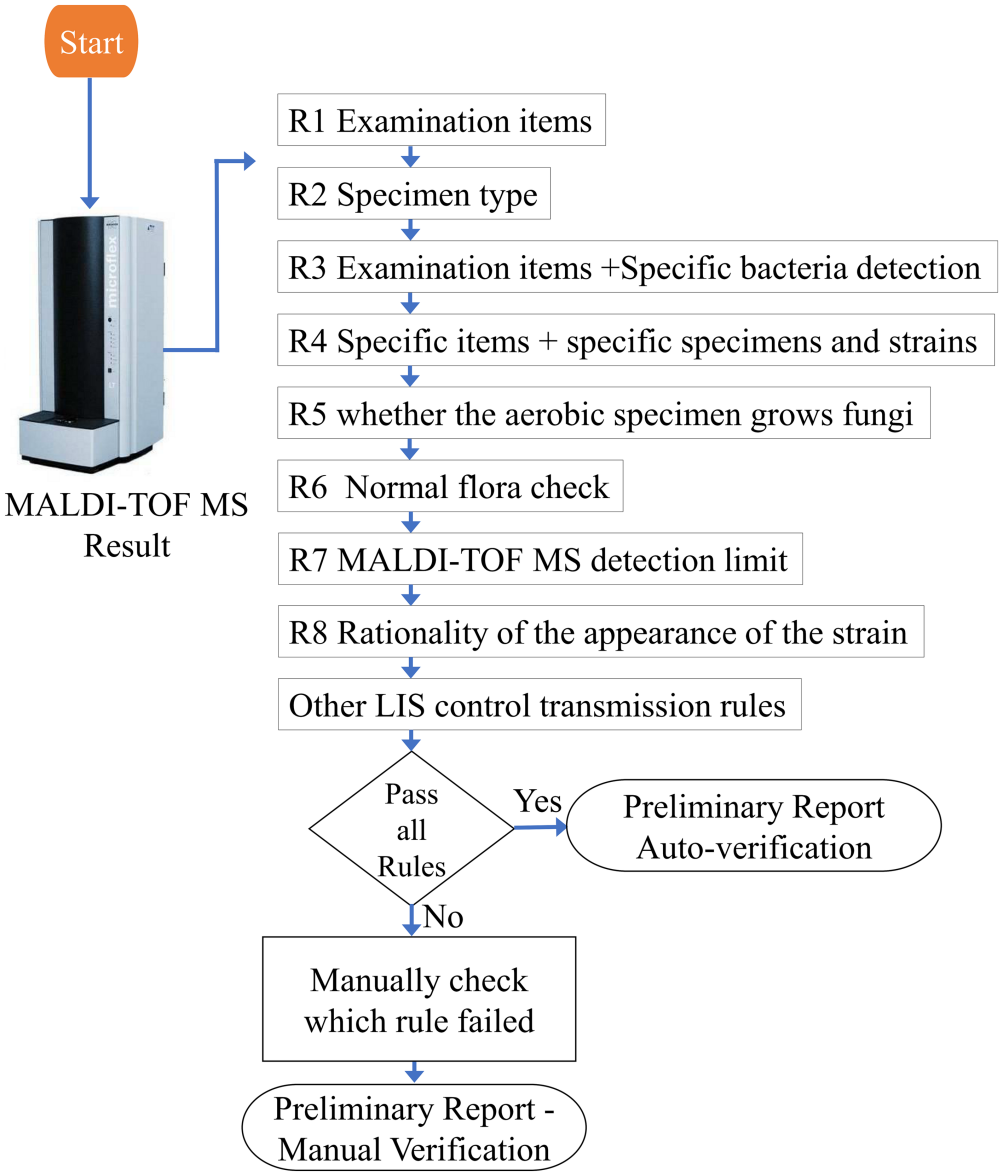
The logic diagram of microorganism auto-verification.

Through the discussion of the principle of auto-verification of bacterial naming by the laboratory technologists specialized in microbiology of our hospital, a total of 9 rules had been formulated based on the inspection items, specimen type, special bacterial screening, the detection limit of mass spectrometer, the rationality of bacterial appearance, the requirement of antibiotic susceptibility test, and so on, which was referenced to CLSI AUTO-10A (Neeley et al., 2006). The logic description was shown in Figure 2.

*Rule 1: Screening for the inspection items, only the specific items that meet the inspection code can be automatically verified and enter the next step*. In our laboratory, medical laboratory scientists may perform identifications for different test items simultaneously during testing operations. For instance, on the same MALDI target plate, there could be identifications for both aerobic culture (code 601) and anaerobic culture (code 603) test items. Therefore, when selecting the auto-verification system (TOF-upload) in Figure 1, the auto-verification system can exclude anaerobic culture (code 603) and proceed with the logic interpretation for aerobic culture (code 601).

*Rule 2: Screening for the category of specimens, the auto-verification only can be operationally used in specific specimens*. For example, in the case of aerobic culture (code 601) with the specimen type labeled as ‘MDR,’ if the purpose of this item is to screen for MDR-*Klebsiella pneumoniae* (multidrug-resistant *Klebsiella pneumoniae*), antibiotic susceptibility test is required to confirm whether it is the target bacterium. Therefore, specimen types of this nature are not suitable for initial bacterial identification issuance. Hence, Rule 2 is designed to exclude certain inappropriate specimen types.

*Rule 3: Verification can only be done for specific items and specific strains*. For example, the report of GBS (*Streptococcus* group *B*) screening in pregnant women can only be verified if GBS (*Streptococcus* group *B*) is identified.

*Rule 4: It can only be verified for specific items and specimens and only for specific strains*. For example, it can only be verified if the doctor’s order is stool culture for *Salmonella* or *Shigella*, and it also is identified as *Salmonella* or *Shigella*.

*Rule 5: Whether there is the growth of fungi in aerobic culture*. If yes, block verification. The laboratory has a policy that if aerobic culture identifies a fungal strain, the specific species name is not directly reported. Instead, it is presented in a generic manner as “yeast-like” in the report. Therefore, auto-verification is not performed for identification results that involve fungal strains. For instance, if aerobic culture (code 601) identifies *Candida albicans*, auto-verification is not conducted, and the report is manually issued as “yeast-like.”

*Rule 6: Normal flora check. For example, sputum samples identified as normal oral flora will be intercepted*.

*Rule 7: MALDI-TOF MS detection limit check*. Due to the detection limit of MALDI-TOF MS, some strains cannot be directly verified, and additional biochemical tests are required to confirm the results.

*Rule 8: Check whether the appearance of the strain selected in the LIS system for the interpretation medium* is consistent with the identification result, if not, it will be intercepted, and then manually verified after confirmation.

*Other LIS control transmission rules*: LIS verification rule check, including the report status that is preliminary (P) or final report (F) inspection and the result of the antibiotic susceptibility test that is already available and is verified as a drug-resistant strain, the report will be intercepted for confirmation.

If all the rules are passed, the auto-verification system will complete the preliminary verification of the bacterial naming. If the report does not meet the rules of auto-verification, the clinical laboratory technologists will confirm the identification result as to which rule is not passed. After the identification result is confirmed to be correct, it will be issued manually.

### 2.4 Auto-verification system validation

After the logic of the auto-verification system was established, the simulated test results were used to track and verify whether the logic setting is correct, and then the bacterial names are automatically verified with clinical samples and the results that were tested in a practical way. After that, the two parts are manually reviewed one by one, (1) Check whether the report issued by the auto-verification system followed the logic and (2) Check whether the reason for auto-verification failure is consistent with the blocking rules. After verification according to the above method, it is confirmed that the correctness of the auto-verification system can be provided for clinical use.

### 2.5 MALDI-TOF MS analysis

The colonies were directly smeared on a MALDI target plate (MSP 96 target ground steel; Bruker Dal-tonics) through a clean toothpick. Each spotted sample was overlaid with 1 μL 70% formic acid and air-dried at room temperature. Add 1 μL of HCCA matrix solution (α-cyano-4-hydroxy-cinnamic acid in 50% acetonitrile – 2.5% trifluoroacetic acid) and air-dry for sure, then use a mass spectrometer [Bruker Microflex LT/SH “smart” MALDI-TOF MS (Bruker Daltonics, Billerica, MA, United States)] with FlexControl software (version 3.4) and MALDI Biotyper (MBT) Compass version 4.1 for analysis and identification. The mass spectrum was acquired in the mass range of 2,000 to 20,000 m/z in linear mode using MALDI-TOF MS for mass detection and analysis of bacterial proteins. The obtained spectrum was analyzed by software and then compared with all the data in the database to obtain microbial identification. The result interpretation standard was that when the identification result score of the test strain was greater than 2.00, the identification reliability was up to the strain name; scores ranging from 1.70 to 1.99 indicated the confidence level of identification up to the genus name of the strain; scores ranging from 0.00 to 1.69 indicated unreliable identification results.

### 2.6 Data analysis

Our laboratory conducts bacterial identification for aerobic specimens every day during regular working hours (8:30–17:00) throughout the year. In this study, the aerobic bacteria culture reports (excluding screening of multi-drug resistant strains and screening of specific strains of stool) from January 2021 to February 2022 were used for calculating the auto-verification pass rate and TAT of the preliminary report. The statistics are as follows:


(1) $$\begin{array}{l} \text{Auto}-\text{verification}\;\text{pass}\;\text{rate}=\\ \frac{\begin{array}{l} \text{total}\;\text{number}\;\text{of}\;\text{reports}\;\text{that}\;\text{were}\;\text{in}\;\\ \text{line}\;\text{with}\;\text{the}\;\text{auto}-\text{verification}\;\text{logic}\; \end{array}}{\begin{array}{l} \text{all}\;\text{strains}\;\text{successfully}\;\text{identified}\;by\;\\ \text{MALDI}-TOF\;MS\;\left(\text{score}\geq 2.0\right)\; \end{array}} \end{array}$$

(2) PreliminaryreportTAT=preliminaryreporttime−receipttime

## 3 Results

### 3.1 Auto-verification pass rate

**Figure 3:**
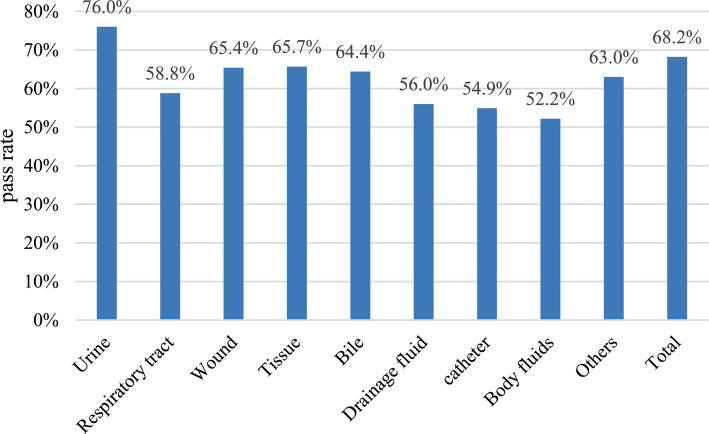
The pass rate of auto-verification for each specimen.

**Figure 4:**
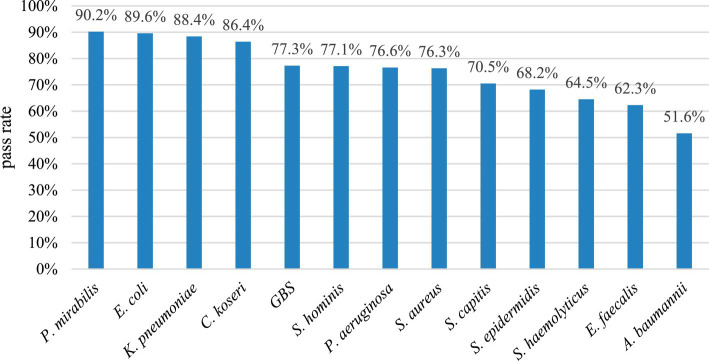
The pass rate of auto-verification for each strain.

According to statistics from January 2021 to February 2022, a total of 70,044 bacterial strains were successfully identified by MALDI-TOF MS in aerobic culture samples, and 47,748 of them passed the auto-verification logic, with a pass rate of 68.2%, which could replace about 2/ 3 of manually issued reports. The auto-verification pass rate of the identification results of urine, respiratory tract specimens, wounds, tissues, bile, drainage fluid, body fluids, and other specimens was 76.0, 58.8, 65.4, 65.7, 64.4, 56.0, 54.9, 52.2, 63.0% (Figure 3). Among them, the auto-verification pass rate of intestinal bacteria was the highest. For example, *Proteus mirabilis* (*P. mirabilis*), *Escherichia coli* (*E. coli.*), *Klebsiella pneumoniae* (*K. pneumoniae*), and *Citrobacter koseri* (*C. koseri*), whose pass rates are all greater than 86%, followed by *Streptococcus* group B (GBS), *Pseudomonas aeruginosa* (*P. aeruginosa*), and *Staphylococcus aureus* (*S. aureus*) were greater than 76% (Figure 4).

### 3.2 Analysis of the reason failed to pass the logic of auto-verification

**Figure 5:**
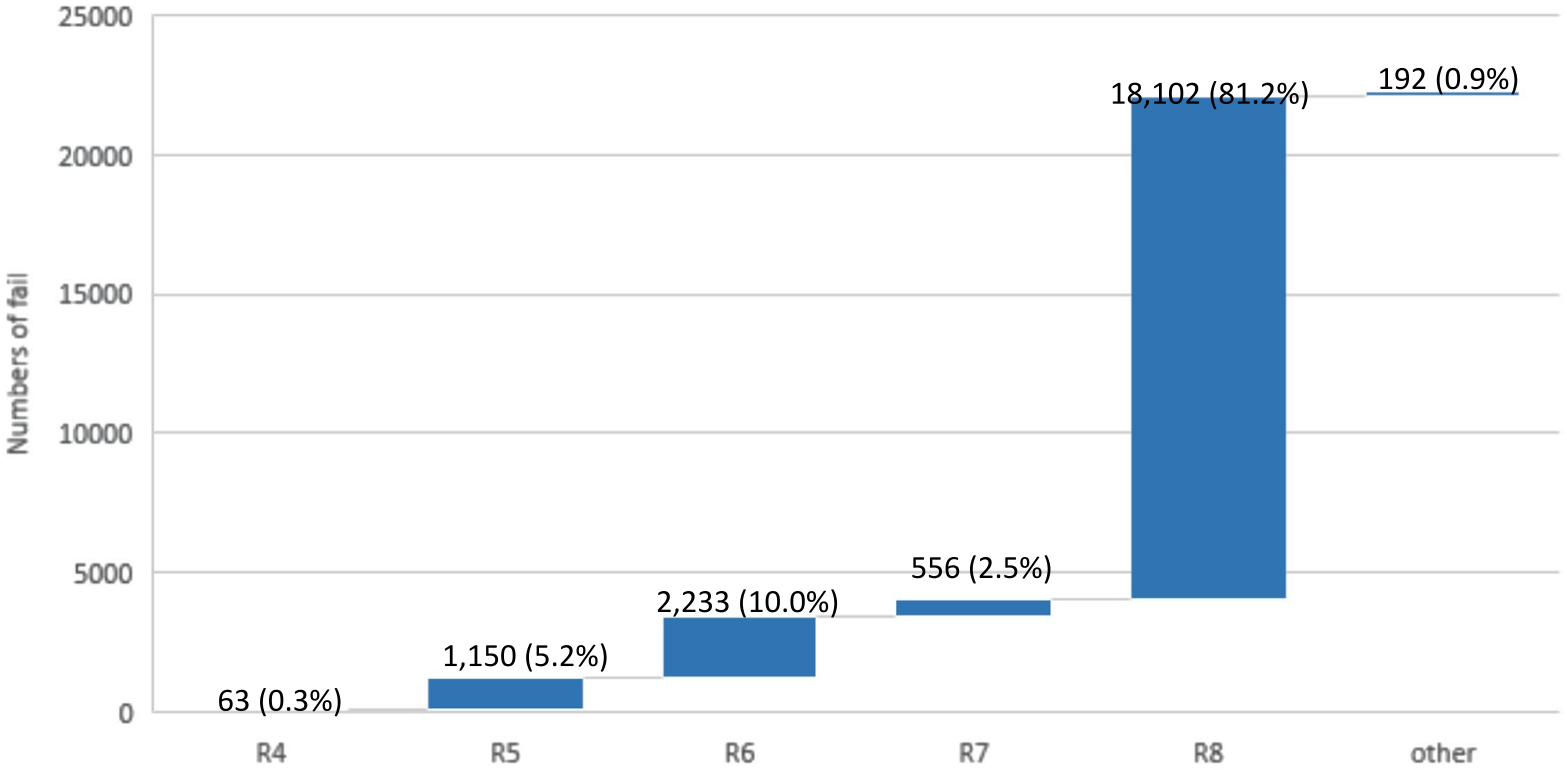
The failure numbers for each auto-verification rule. The data labels indicate the number of failures, with the percentage of total failures in parentheses. The total number of failures is 22,296.

A total of 22,296 identification results failed the auto-verification (Figure 5). There were 1,150 results (5.2%) that failed to pass Rule 5, where the identification results were mainly yeast, which did not need to provide the name of the strain. According to the policy, when gram stain distinguishes a “yeast-like” appearance or MALDI-TOF identifies *Candida* spp. and meets the reporting criteria, the laboratory issues a report indicating “yeast-like” for aerobic culture results to the clinical end. The report includes a note stating that if further identification is needed, a separate fungal culture test request should be submitted for subsequent identification. 2,233 results (10%) failed to pass Rule 6, where the identification results were mainly normal flora in the respiratory tract (including sputum, bronchial washing, and bronchial-alveolar lavage) and urine, which did not need to be reported. Sputum commonly contains *Coagulase (−) Staphylococcus* (CoNS), *Corynebacterium* spp., *Candida* spp., α,γ*-Streptococcus*, (excluding *N. meningitidis* and *N. gonorrhoeae*), *Haemophilus* (excluding *H. influenzae*), *Moraxella* (excluding *M. catarrhalis*), *Rothia*, etc., which do not require identification. 556 results (2.5%) failed to pass Rule 7, where the main strains included *Streptococcus pneumoniae* (*S. pneumoniae*)/ *Streptococcus mitis* (*S. mitis*) or *Aeromonas hydrophila* (*A. hydrophila*) complex /*Aeromonas caviae* (*A. caviae*) complex, etc., which could only be distinguished by further biochemical reactions. The largest number of identification results failed to pass Rule 8, with a total of 18,102 results (81.2%). The reason was that the identification results were not consistent with the rationality of the appearance of the strain. When the clinical laboratory technologists suspect that there is another strain or are not sure about the species to be identified, they deliberately do not select the appearance or select a special mark to block auto-verification or select the wrong appearance of the strain. If it failed to pass the auto-verification logic, the clinical laboratory technologists would check it one by one, and then issue it manually after confirming that the identification result was correct.

### 3.3 Preliminary report turnaround time (TAT)

**Figure 6:**
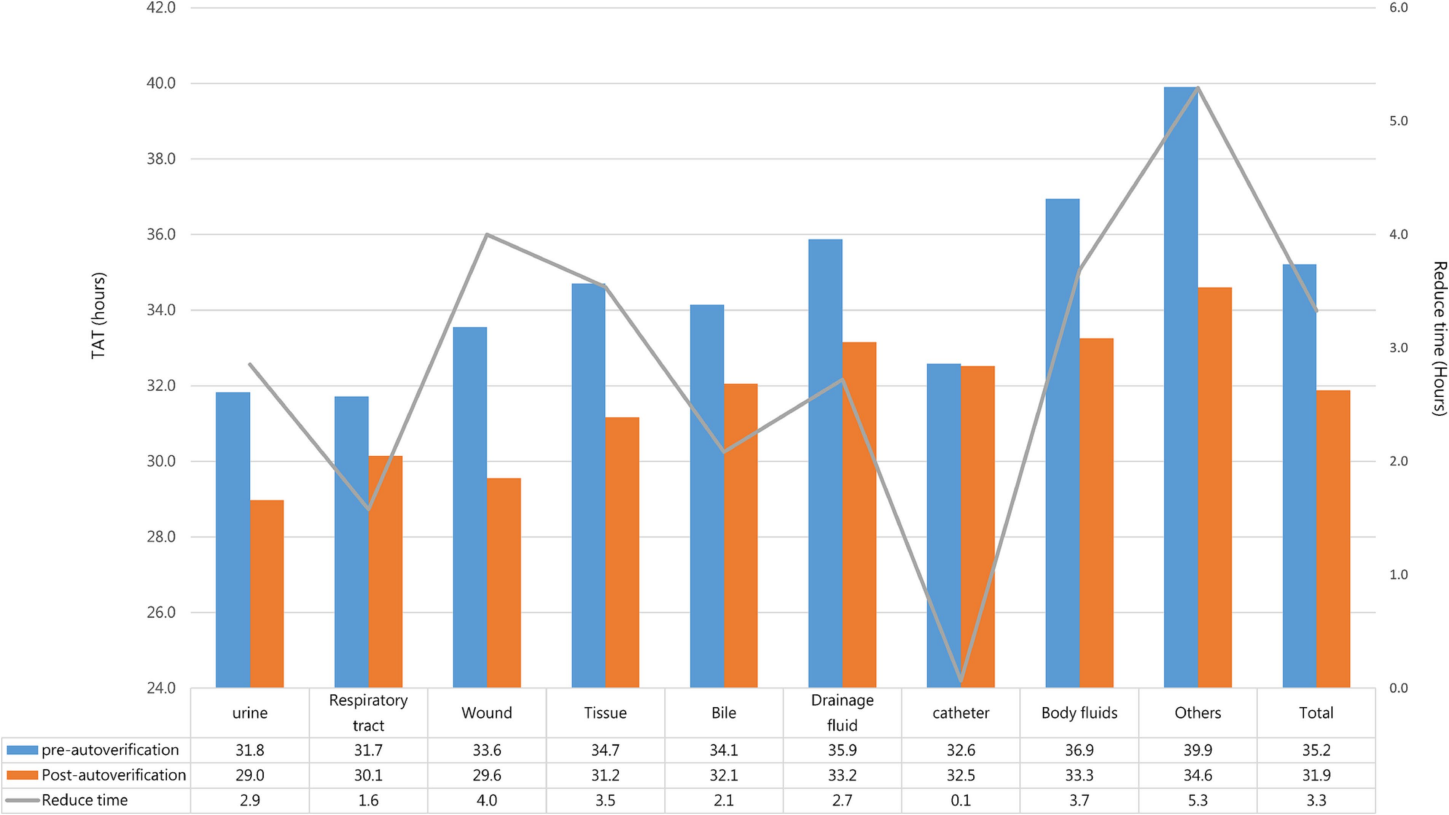
Preliminary report TAT for each specimen.

The total average preliminary report TAT through auto-verification was 31.9 h, which was about 3.3 h less than the average of 35.2 h before auto-verification was used. In addition, it could be found that the TAT of the wound could be reduced from 33.6 h to 29.6 h, a decrease of 4 h; the body fluid could be reduced from 36.9 h to 33.3 h, a decrease of 3.7 h; the tissue could be reduced from 34.7 h to 31.2 h, a decrease of 3.5 h (Figure 6). The TAT reduction time of the above three types of samples was better than the overall report reduction time after using auto-verification. However, although the TAT improvement rate of urine was not better than the overall average, its TAT was the shortest among all samples. Because the bacteria growth in urine is relatively simple, most of which are *Enterobacteriaceae* that grows fast and can grow into mature colonies for identification in a short period. Therefore, it only took about 29 h for the urine specimen to provide the preliminary report. For the other part, specimens such as the respiratory tract, bile, and catheter could reduce the TAT by about 0.1 to 2.1 h.

**Figure 7:**
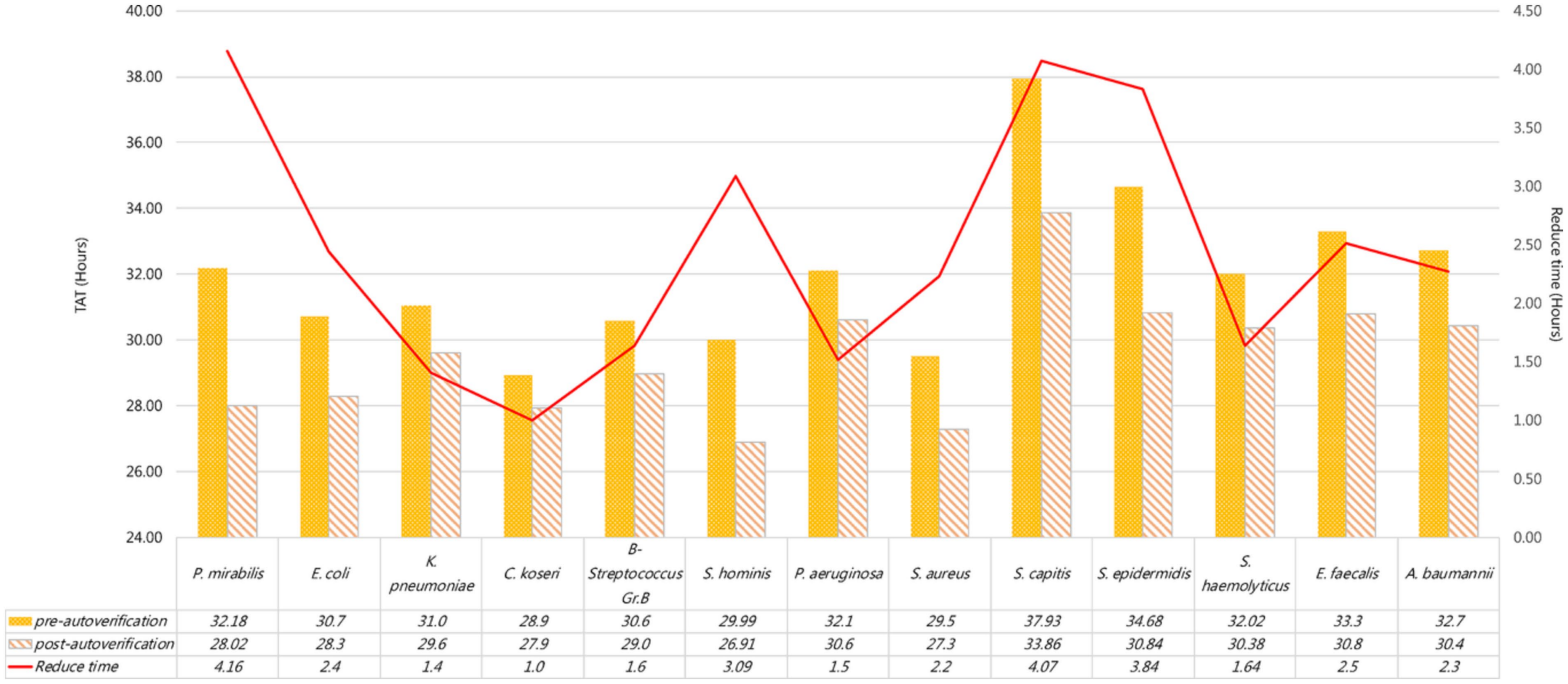
Preliminary report TAT for each strain.

Further analysis of the TAT of common strains, including *P. mirabilis*, *K. pneumoniae*, *C. koseri*, and *E. coli* is the most common among enterobacteria (Supplementary Table S2), its TAT decreased 2.4 h after using auto-verification, while *K. pneumoniae*, *C. koseri* decreased by 1.4 and 1 h, respectively, while *S. aureus*, *P. aeruginosa*, and GBS decreased by 2.2 h, 1.5 h, and 1.6 h, respectively. Among the common strains, CoNS had the greatest improvement. For example, *Staphylococcus capitis* (*S. capitis*) and *Staphylococcus epidermidis* (*S. epidermidis*) both reduced TAT for nearly 4 h (Figure 7).

## 4 Discussion

The work of a traditional microbiological laboratory is very time-consuming. From the inoculation of the specimen to the appropriate medium, the colony interpretation and identification can only be carried out after 18–24 h of bacterial growth. If there is no mass spectrometer or other automated identification system in the laboratory, the biochemical reaction test can be carried out for additional 18–24 h. It often takes more than 36–48 h to obtain the identification results of a bacterium. Although the time required for identification has been significantly reduced after the popularization of automated instruments such as mass spectrometers (Dingle and Butler-Wu, 2013), it is often necessary to rely on manual and time-consuming checks one by one after the identification results of the mass spectrometer are obtained before issuing a preliminary bacterial name report. The auto-verification of bacterial identification through MALDI-TOF has not been explored or developed by other researchers. This is because the bacteriological testing process requires a high level of expertise and judgment to provide the clinical setting with accurate and appropriate reports. Therefore, this study designed an auto-verification system against the microbiological laboratory of our hospital to assist in the preliminary report verification of bacterial names to increase efficiency and improve TAT.

Clinical microbiological specimens exhibit complex growth patterns, with testing items categorized into aerobic culture, anaerobic culture, or fungus culture. The use of specific culture medium and conditions for each testing item has been established from the initial stages. Therefore, in this study, the calculation of TAT for the auto-verification of aerobic bacterial identification was not affected by mixed infections. The research results showed that the auto-verification system developed by our hospital could replace about 2/3 of the manual verification and issuance of reports, among which the auto-verification of urine specimens had the highest pass rate. We suggested that it may be because most of the intestinal bacteria in the urine are easier to identify so it was easier for laboratory technologists to choose the correct appearance when judging the appearance of bacteria.

There are many inspection items in the microbiology laboratory, and the meanings of bacteria grown from different specimens are also different. Therefore, there are many limitations in the logical design, including test items, test types, special bacteria screening, detection limitation of the mass spectrometer, appearance rationality of strains, and the requirement of antibiotic susceptibility testing. Among them, the most important factor affecting the pass rate of auto-verification is whether the selection of strain appearance is reasonable. During the interpretation of culture plates by medical laboratory scientists, the initial step is to identify gram-negative or gram-positive bacteria for suspected potential pathogens, then further observe and judge the appearance of the strain. Macroscopic observation of colonies is a crucial step for medical laboratory scientists because it allows for preliminary screening of microorganisms. Experienced personnel may even directly recognize colony morphology to make educated guesses about the bacterial species. For example, when the suspected strain in a specimen is gram-negative bacteria and appears to be *Enterobacteriaceae*, the medical laboratory scientist would choose ‘G (−)’ in the ‘Gram stain’ field and select the appropriate appearance in the ‘Appearance’ field. Different specimen categories have different antibiotic susceptibility testing modules, so there are corresponding choices for ‘Appearance’ based on the specimen type. For instance, for *Enterobacteriaceae* in urine samples, one may choose “U-m” or “U-L+” or “U-L-” or “U-pro,” while for *Enterobacteriaceae* in non-urine samples, one may choose “m” or “L+” or “L-” or “pro.” If there is suspicion of *S. aureus* in a wound specimen, the ‘Gram stain’ field would be selected as G (+), and in the ‘Appearance’ field, “Sa” would be chosen. For CoNS, “STA” would be selected. Conversely, if the strain is *A. baumannii*, and the medical laboratory scientist selects “U-m” or “m” in the ‘Appearance’ field, it cannot be automatically verified because “U-m” and “m” are appearance settings only suitable for *Enterobacteriaceae* strains. Additionally, samples may be susceptible to contamination, especially respiratory specimens that may be influenced by upper respiratory tract flora (Jourdain et al., 1997), and urine specimens that may be affected by contamination risk factors such as obesity, female gender, and pregnancy (Hansen et al., 2022), leading to inaccuracies in the automated verification system. For instance, distinguishing between infection and pre-existing colonization of *A. baumannii* in respiratory specimens can be challenging. The mere cultivation of *A. baumannii* from a sample does not universally indicate an active infection. This remains a contentious issue (Feng et al., 2022).

Another species commonly associated with the incorrect selection of appearance is *Enterococcus faecium* (*E. faecium*). Typically, when medical laboratory scientists suspect the colony to be *E. faecium*, they would choose the growth appearance “NB.” This growth appearance corresponds to five antibiotic modules for antibiotic susceptibility testing, and if *E. faecium* is vancomycin-resistant, additional second-line antibiotic susceptibility testing results will be issued. However, in some cases, when medical laboratory scientists reviewing the culture plates discover a previous cultivation of vancomycin-resistant *Enterococcus* in the patient’s specimen, they often switch the growth appearance from “NB” to “VRE.” This growth appearance corresponds to susceptibility testing for seven antibiotics (including second-line antibiotics), but it does not align with the logic of auto-verification, leading to a lower auto-verification pass rate. In the future, it may be considered to include the growth appearance “VRE” in the auto-verification logic to enhance the auto-verification pass rate for *E. faecium*.

The appearance of some bacteria is quite variable. For example, *E. coli* has many different types (Barcella et al., 2016). Therefore, when the laboratory technologist suspects that there are two kinds of *E. coli* with different appearances, he will select special marks for the appearance of the second one to intercept auto-verification, such as U-m “*.” This is done to prevent confusion for clinicians when encountering the report with the same bacterial name from two strains. Therefore, the ‘Appearance’ field involves selecting different categories for suspected bacterial strains, and these category choices also impact the drug modules for subsequent antibiotic susceptibility test. The correspondence rules between the ‘Appearance’ field and the colonies of various bacterial strains have been set as part of the logic for Rule 8. If the set logic is satisfied, the rule is passed; otherwise, selecting the wrong appearance will result in the system not passing judgment.

Another common rule for auto-verification failure was Rule 6. According to the definition in our laboratory manual, common normal oral flora in sputum includes CoNS, *Candida* spp., α,γ*-Streptococcus* (excluding *N. meningitidis* and *N. gonorrhoeae*), *Haemophilus* (excluding *H. influenzae*), *Moraxella* (excluding *M. catarrhalis*), *Rothia*, etc. Common potential pathogens encompass *S. pneumoniae*, *S. aureus*, *Moraxella catarrhalis* (*M. catarrhalis*), *Haemophilus influenzae* (*H. influenzae*), *Neisseria meningitidis* (*N. meningitidis*), *Streptococci* Group B, C, and G, *Pasteurella* spp., *Enterobacteriaceae*, *Stenotrophomonas maltophilia*, *Acinetobacter* spp., *P. aeruginosa*, *Burkholderia cepacian* (*B. cepacia*), and other aerobic gram-negative bacilli. Such potential pathogens may exist in small quantities in the sputum of normal individuals and can cause infection when present in large quantities. Therefore, as per the manual, identification and antibiotic susceptibility test are only performed when the colony count of these potential pathogens exceeds the usual bacterial quantity in the respiratory tract. Medical laboratory scientists, while interpreting culture plates, rely on experience to determine whether a potential pathogen is predominant or if there are suspicious colonies that require identification. Upon analysis, the main reason for Rule 6 not passing was the identification of CoNS, *Corynebacterium* spp., *Candida* spp., α,γ*-Streptococcus*, etc., which are normal oral flora, during the judgment of whether a potential pathogen is present. This leaded to a failure in auto-verification.

Considering the above reasons, one of the major factors affecting the pass rate of auto-verification was the ability of laboratory technologists to recognize bacteria and whether they follow the logic system of auto-verification when using the LIS system. Inexperienced laboratory scientists often struggle to choose the correct ‘Appearance’ or may not choose an appearance at all. Instead, they wait for the MALDI-TOF to identify the bacterial name before making appearance selections and manually issuing reports. They may also select normal flora for identification, which does not require reporting, leading to a failure in Rule 6. However, this study did not analyze the colony-interpretation ability of new personnel. This aspect could serve as an indicator of bacterial recognition ability and might be focused on during the training of new personnel to enhance their ability to identify colonies and, consequently, improve the pass rate of auto-verification.

Our laboratory uses MALDI-TOF to issue reports. Only when the first and second place bacteria names in the identification results have the same name and the first-place score ≥ 2.0 can the bacteria be released. However, some species, such as *Acinetobacter baumannii* (*A. baumannii*) and *Acinetobacter nosocomialis* (*A. nosocomialis*), have highly similar protein spectra, making it challenging for the mass spectrometer to clearly differentiate them. Often, the identification results show that the first and second place bacteria names belong to the same genus but different species. For instance, *A. baumannii* and *A. nosocomialis* may appear in either the first or second position, making it difficult to distinguish between them. As a current practice, we reprepare the sample and conduct a re-identification. If the results still do not meet the criteria for reporting the species, we manually report it as the *A. calcoaceticus*-*A. baumannii* complex (ACB complex). Since the first and second names are different in this case and do not meet the reporting criteria, these instances are not included in the statistics. However, in the future, it might be considered to systematically modify the LIS system to improve the identification success rate for *Acinetobacter* species.

**Figure 8:**
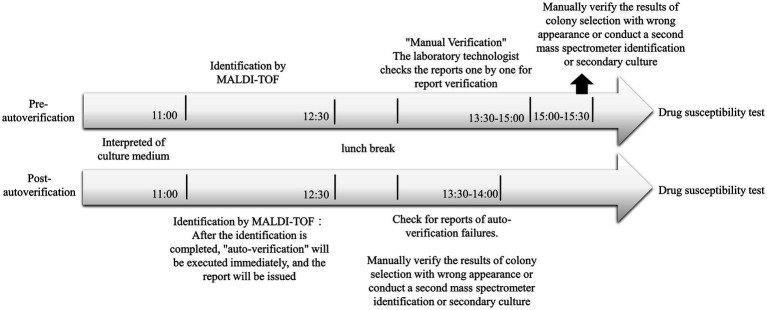
Schematic diagram of manual verification and automatic verification process.

The biggest difference between manual verification and auto-verification in the reporting process is the process after the completion of identification (Figure 8). When the manual verification process is carried out, the time for the mass spectrometer to complete the verification is about 12:30 p.m., so the laboratory technologist often completes the verification report one by one after returning from lunch break, and this part will be slightly delayed due to the different processes of each personnel. After that, the strains that cannot be reported will be identified for the second time or the next day after the second culture. The automatic verification process is that after the mass spectrometer identification is completed, the personnel can directly click the “TOF-upload” to upload the identification results immediately. According to the research results, about 2/3 of preliminary report verification of the bacteria name could be completed before the lunch break. After the lunch break, the strains that cannot be issued a report will be identified or cultured for the second time. Therefore, the time for issuing the report using the auto-verification system will be from about 15:00 to about midday to complete. After such improvement, the TAT for preliminarily reporting the bacterial name was shortened from 35.2 h to 31.9 h on average. Due to the use of the auto-verification system, medical laboratory scientists no longer need to manually verify each report. They only need to check if a second identification is required. As a result, each laboratory scientist can save approximately half an hour of working time, contributing to increased efficiency.

On the other hand, a study has pointed out that laboratory automation could reduce the reporting time of positive blood cultures and improve the management of patients with blood flow infections (De Socio et al., 2018). In the future, the application of the auto-verification system to the blood culture process in our laboratory can be assessed to enhance the TAT for blood culture reports. Additionally, gaining early insights into the bacteria causing infections and their potential resistance or susceptibility can aid in rapidly identifying the source of infection. This, in turn, can optimize and determine the best course of antibiotic treatment at the appropriate time, directly impacting patient outcomes and healthcare costs (Bassetti et al., 2022). Before the microbiology laboratory becomes a “total laboratory automation (TLA),” the future auto-verification system will continue to be optimized. It is anticipated that auto-verification will be introduced for positive results in blood culture bacterial identification or antibiotic susceptibility test. There will also be a focus on enhancing the education and training of medical laboratory scientists in colony recognition, along with ongoing improvements to the auto-verification system, aiming to improve the current pass rate of only 68.2%. Although the microbiology laboratory has introduced automated machines for microbial identification and antibiotic susceptibility test, there are still many manual steps involved. Further optimization of the verification system is expected to provide a better platform for clinical testing.

## 5 Conclusion

Although our institution has utilized an auto-verification system to reduce the TAT by approximately 3.3 h, providing an early reference for antibiotic use, the actual turnaround time for antibiotic susceptibility test has not improved significantly. Despite the shortened TAT for medical laboratory scientists on the day of testing, it still takes 18 to 24 h of incubation time to complete the final reporting process after the antibiotic susceptibility test is performed. The laboratory is currently working towards the goal of implementing an automatic verification system for antibiotic susceptibility test, aiming to advance the automation process of microbial reporting and enhance the overall efficiency of microbiological test reporting in the future.

## Data availability statement

The raw data supporting the conclusions of this article will be made available by the authors, without undue reservation.

## Author contributions

Y-HO: Data curation, Writing – original draft. Y-TC: Formal analysis, Writing – original draft. D-PC: Validation, Writing – review & editing. C-WC: Methodology, Writing – original draft. K-CT: Methodology, Writing – original draft. C-HW: Methodology, Writing – original draft. A-JK: Methodology, Writing – original draft. H-LY: Methodology, Writing – original draft. C-GH: Conceptualization, Writing – original draft.
