# Supplementary material for: Benefit analysis of the auto-verification system of intelligent inspection for microorganisms
Source: Front Microbiol. 2024 Mar 18;15:1334897. doi: 10.3389/fmicb.2024.1334897 (PMC10982382; doi:10.3389/fmicb.2024.1334897)
Supplement: Supplementary file 1 [file Data_Sheet_1.docx]

Supplementary Table 1. The pass rate of auto-verification for each specimen

|  | N^*^ | Pass | Fail | Pass rate |
| --- | --- | --- | --- | --- |
| Urine | 29,052 | 22,087 | 6,965 | 76.00% |
| Respiratory tract | 6,977 | 4,101 | 2,876 | 58.80% |
| Wound | 19,675 | 12,873 | 6,802 | 65.40% |
| Tissue | 2,963 | 1,948 | 1,015 | 65.70% |
| Bile | 1,491 | 960 | 531 | 64.40% |
| Drainage fluid | 1,319 | 739 | 580 | 56.00% |
| catheter | 1,226 | 673 | 553 | 54.90% |
| Body fluids | 2,393 | 1,249 | 1,144 | 52.20% |
| Others | 4,948 | 3,118 | 1,830 | 63.00% |
| Total | 70,044 | 47,748 | 22,296 | 68.20% |

*N= Total number of identifications (MALSI-TOF MS score≥2.0)

Supplementary Table 2. The pass rate of auto-verification for each strain

|  | Total | Pass | Pass rate |
| --- | --- | --- | --- |
| *Proteus mirabilis* | 2108 | 1902 | 90.2% |
| *Escherichia coli* | 16860 | 15106 | 89.6% |
| *Klebsiella pneumoniae* | 3298 | 2916 | 88.4% |
| *Citrobacter koseri* | 828 | 715 | 86.4% |
| *Streptococcus* group B | 1325 | 1024 | 77.3% |
| *Staphylococcus hominis* | 896 | 691 | 77.1% |
| *Pseudomonas aeruginosa* | 5175 | 3963 | 76.6% |
| *Staphylococcus aureus* | 6634 | 5063 | 76.3% |
| *Staphylococcus capitis* | 905 | 638 | 70.5% |
| *Staphylococcus epidermidis* | 3946 | 2690 | 68.2% |
| *Staphylococcus haemolyticus* | 1663 | 1072 | 64.5% |
| *Enterococcus faecalis* | 3842 | 2392 | 62.3% |
| *Acinetobacter baumannii* | 1452 | 749 | 51.6% |

Supplementary Table 3. The failure rate for each auto-verification rule

|  | R4 | R5 | R6 | R7 | R8 | other | Total |
| --- | --- | --- | --- | --- | --- | --- | --- |
| Fail | 63 | 1,150 | 2,233 | 556 | 18,102 | 192 | 22,296 |
| Failure rate | 0.3% | 5.2% | 10.0% | 2.5% | 81.2% | 0.9% | 100% |
